# Molecular advances in transfusion medicine: a narrative review

**DOI:** 10.3389/fmed.2025.1607340

**Published:** 2025-09-29

**Authors:** Wjdan A. Arishi, Ahmed Yaqinuddin, Muhammad Raihan Sajid

**Affiliations:** College of Medicine, Alfaisal University, Riyadh, Saudi Arabia

**Keywords:** narrative review, transfusion medicine, genotyping, molecular diagnostics and therapeutic approaches, CRISPR

## Abstract

This narrative review reports recent advances in transfusion medicine, encompassing enhancements in molecular diagnostics, blood engineering, and therapeutic technologies. It summarizes findings from peer-reviewed studies relevant to these emerging areas. Molecular diagnostics have evolved from low-throughput polymerase chain reaction (PCR) -based methods with specificity for known polymorphisms to high-throughput approaches, such as microarray-based genotyping and next-generation sequencing, which enable the detection of both established and novel blood group variants. In addition, the integration of genomic data with serological testing has improved the accuracy of blood group profiling and enhanced donor screening for rare antigens. Advances in blood engineering are demonstrated by studies employing induced pluripotent stem cell reprogramming and clustered regularly interspaced short palindromic repeats (CRISPR)-mediated gene editing to produce red blood cells engineered for multiple rare or null antigen phenotypes. Other investigations describe noninvasive fetal RHD genotyping (Rhesus D antigen gene genotyping), recombinant DNA technologies for standardized reagents, and gene therapy approaches that extend clinical applications beyond diagnosis to treatment. Collectively, this review demonstrates that a diversified use of molecular, genomic, and cellular technologies is reshaping antigen matching and therapeutic strategies in transfusion medicine.

## 1 Introduction

Blood transfusion practice has undergone significant evolution, transforming from a high-risk intervention into a critical component of modern healthcare (1). Early transfusions, one of the first forms of personalized medicine, involved a direct connection between donor and recipient in the same room (2). The introduction of the ABO blood group system in 1900 by Karl Landsteiner transformed whole-blood transfusion practice, with ABO blood group compatibility becoming the cornerstone of safe transfusion (3).

Transfusion medicine encompasses more than blood transfusion alone and plays a significant role in immunohematology, transplantation, cellular therapy, and disease management (4). The blood group antigens on the surface of all red blood cells (RBCs) are responsible for cellular recognition and the immune response (5). According to the International Society of Blood Transfusion (ISBT), these antigens are grouped into 48 systems comprising more than 360 recognized antigen specificities (5). The ABO and Rhesus (Rh) systems are the most clinically relevant, affecting transfusion compatibility, hemolytic reactions, and disease susceptibility (3). New molecular technologies have broadened our knowledge of these systems, providing information on their genetic and structural basis (6).

In the last couple of decades, cutting-edge molecular, cellular, and proteomic technologies have been integrated into transfusion medicine (7). These include next-generation sequencing (NGS), single-cell RNA sequencing, and proteomics, which have transformed diagnostic and therapeutic approaches (8). The emerging era of gene-editing and omics technologies has broadened the horizons of precision medicine and personalized strategies to address the problems of alloimmunization, rare phenotypes, and transfusion-related complications (9, 10).

This review discusses innovations in transfusion medicine, including the role of blood group antigens and advancements in molecular diagnostics, blood engineering, and therapeutic technologies (11, 12).

## 2 Molecular diagnostic advances

### 2.1 Polymerase chain reaction (PCR-based) genotyping

Various PCR-based methods have increased the accuracy of blood group typing and effectively resolved certain complications of conventional serological methods (13). These include conventional PCR, real-time PCR (qPCR), multiplex PCR, and digital droplet PCR (ddPCR). These methods utilize designed primers specific to known alleles to amplify DNA fragments, Then the results are analyzed through gel electrophoresis or restriction enzyme digestion (13). Among these, real-time PCR enables the rapid and accurate identification of blood group antigens by detecting polymorphism responsible for antigen expression (13). This technique uses fluorescently labeled probes designed to bind specific DNA sequences. During amplification, Taq polymerase cleaves the probe, releasing a fluorescent signal that is proportional to the amount of target DNA, thereby enabling both qualitative detection and quantitative analysis (14). The high sensitivity and specificity of this method make it well-suited for genotyping applications (14, 15).

Multiplex PCR has been used to simultaneously amplify several target DNA sequences in a single reaction by employing multiple primer sets. This approach facilitates the detection and analysis of multiple genes or genetic variations, saving time, reagents, and effort compared to performing separate individual reactions (16). In this method, two reaction mixtures, containing allele-specific primers and the DNA fragments for different blood group antigens (Duffy, Kidd, Rh, Diego, and MNS) are amplified (17). The technique has been validated against standard serology and polymerase Chain Reaction-Sequence Specific Primer (PCR-SSP) and proved to be valid and suitable for supplying antigen-matched units to transfusion-dependent patients (17).

Digital droplet PCR (ddPCR) is another sensitive and specific technique used in blood group genotyping. Unlike traditional PCR, wherein the amplification is done on bulk DNA, ddPCR allows for partitioning a sample into thousands or millions of nanodroplets, by which DNA can be individually amplified and quantified on a single-molecule basis (18). This method enables the detection of low-frequency variants and minor alleles, making it especially useful for transfusion-dependent populations (19). ddPCR has the advantage of accuracy in mixed populations, effectively solving challenges associated with multiply transfused patients (19).

### 2.2 Matrix-assisted laser desorption/ionization time-of-flight mass spectrometry (MALDI-TOF MS) + PCR-SSP

MALDI-TOF MS is a highly accurate analytical technique that is used to measure the mass of DNA fragments. In this method specific primers will bind to the polymorphic site and will be extended by DNA polymerase by adding one or a few nucleotides complementary to the target sequence. Then the amplified extension products are ionized by laser, electrostatically accelerated, and separated based on their mass-to-charge ratio (m/z), facilitating accurate differentiation between variants (20). To improve reliability, it is often combined with PCR-SSP, a targeted approach in which primers amplify DNA only when a specific allele is present. The integration of MALDI-TOF with PCR-SSP, provides a powerful strategy for identifying both common and rare blood group variants (21).

### 2.3 Transfusion medicine array

In transfusion medicine, Microarray provides the molecular resolution needed to define blood group antigens, thus assessing transfusion compatibility, minimizing adverse reactions, and enhancing transfusion safety (22). Single Nucleotide Polymorphisms (SNPs), defined as single-base variations in the DNA sequence, and can be detected using SNP array-based genotyping. In this method, DNA is hybridized to immobilized oligonucleotide probes on chips allowing detection of multiple SNPs simultaneously. This technique is valuable in cases where serological methods are inconclusive or unavailable. However, most commercially available SNP arrays are limited by the number of loci and often fail to capture many transfusion-relevant variants (22). To overcome these challenges, a specialized genome-wide SNP array, called the transfusion medicine array (TM-Array), was developed using the Affymetrix Axiom platform to facilitate the comprehensive study of both common and rare transfusion-associated variants across genetically diverse donor and recipient populations. The TM-Array includes probes with targets involved in RBC and platelet structure and function, the human leukocyte antigen (HLA) system, and blood groups, including human platelet antigen (HPA) (22). The Affymetrix Axiom platform is a hybridization-based genotyping array that offers a flexible choice of predesigned modules and customizable content. The TM-Array features approximately 1.38 million hybridization probes, representing about 879,000 SNPs and copy number polymorphism markers (22). The array demonstrated exceptional technical performance, with over 99% of SNPs reliably genotyped, as well as strong reproducibility, showing an error rate below 0.03%. It also exhibited excellent parent–child trio accuracy of 99.97%. Blood group genotyping results were in concordance with serological findings, and the array could detect rare alleles with a minor allele frequency as low as 0.5% (22).

### 2.4 NGS

NGS has been utilized for analyzing genome variations, including SNPs, structural variations, and copy number variations (23). NGS has the advantage of enabling the complete genotyping of many blood group systems in a single test, allowing for the detection of both common and rare variants (23). NGS has successfully characterized complex genomic configurations in systems like Rh and MNS, which are often difficult to interpret using conventional methods (24). By utilizing high-throughput sequencing technologies, NGS facilitates complete analysis of the entire blood group gene loci, improving accuracy and resolution in transfusion-related genotyping (25). In the context of NGS-based genotyping, massively parallel sequencing, also known as high-throughput sequencing, has been used for profiling full blood group genotypes, allowing for the identification of known and novel genetic variants affecting blood group antigens (26). This sequencing method is based on fragmenting genomic DNA, then sequencing it in parallel and aligning the resulting reads to a reference genome for variant detection (26). This approach enables the discovery of novel polymorphisms that cannot be detected by microarray or traditional genotyping methods (26). Short-read NGS has been applied to whole-genome blood group genotyping (27). For instance, the Ion Torrent system, employing NGS technology, detects nucleotide incorporation by measuring pH changes during DNA synthesis (28). This allows for real-time and label-free sequencing. It provides reads with an average insert size of 200 base pairs, suitable for targeted sequence amplification and the sequencing of specific genome regions, enabling an efficient and cost-effective focus on clinically relevant variants (28). Whole-genome sequencing, used for the genetic analysis of variants associated with the Kidd, Duffy, and Kell blood group systems, was performed by generating 150 bp paired-end reads with 30X coverage depth employing Illumina technology, thus revealing all known and novel variants across the genome (29). These findings highlight the potential of NGS technologies to improve transfusion safety through more accurate and individualized blood group genotyping (29).

### 2.5 Emerging CRISPR diagnostics

A CRISPR/Cas13a-based SNP was developed to detect specific ABO types, allowing for the resolution of weak and subgroup alleles. Unlike the widely known CRISPR/Cas9 gene-editing system which uses Cas9 endonuclease to target DNA, the CRISPR/Cas13a system targets RNA molecules. In this method, DNA from the ABO gene was amplified using PCR. Then the amplified DNA was transcribed into RNA, making it a target for Cas13a. A specifically designed guide RNA (gRNA) was used to recognize the RNA sequence with the SNP site, the Cas13a activates and cleaves the RNA target. This cleavage will produce fluorescent signal that confirms the presence of the SNP. This approach demonstrated accurate ABO genotyping with a sensitivity of approximately 50 pg. per reaction and a detection time of 60 min (30).

### 2.6 Clinical applications of molecular diagnostics

#### 2.6.1 Hemolytic Disease of the Fetus and Newborn (HDFN)

Hemolytic Disease of the Fetus and Newborn (HDFN) is an immune-mediated condition that develops when maternal immunoglobulin G (IgG) antibodies produced against fetal RBCs cross the placenta and cause hemolysis of fetal RBCs (31). Antibodies against RhD antigen are the most clinically significant compared to other blood group systems such as Kell, Duffy, Kidd, and ABO (31). Molecular diagnostics contribute to the non-invasive prediction and diagnosis of HDFN. Real-time PCR using cell free fetal DNA to detect fetal RHD, KEL, and ABO genotypes, as demonstrated by Song et al. (32) where they combined TaqMan and SYBR PCR in fetal ABO typing showing accuracy rate of 93.2%. Similarly, multiplex PCR-SSP used for screening of RHD variants, particularly in regions with high prevalence of DEL alleles, where serology encounters limitations (32). ddPCR has expanded these capabilities, with O’Brien et al. (31) showing its utility to predict fetal inheritance of KEL1, Duffy, or Rh antigens causing risk of fetus alloimmunization. These examples illustrate how PCR-based techniques are used for early prediction and diagnosis of HDFN, reducing the need for invasive procedures such as amniocentesis (31). Targeted next-generation sequencing (NGS) has also been applied in the identification of novel antigens associated with HDFN. As an example, exome sequencing of a three-generation pedigree revealed previously unrecognized low-frequency red cell antigen, provisionally named SARAH (MNS47), which was linked to a G240T single-nucleotide variant in the GYPA gene. This finding reinforced the role of NGS in discovering rare antigens with clinical significance in transfusion medicine (33). In addition, targeted NGS has been used for non-invasive fetal ABO blood group prediction in type O pregnancies by analyzing cell-free fetal DNA from maternal plasma. This technique identified both common and rare ABO variants, highlighting its utility in reducing the risk of HDFN in alloimmunized pregnancies and offering a safer alternative to invasive diagnostic procedures (25).

#### 2.6.2 Transfusion compatibility

Achieving reliable transfusion compatibility is vital for safe transfusion, yet traditional serological tests can become unreliable, especially in patients with autoimmune hemolytic anemia (AIHA), those who have received multiple transfusions, or individuals with rare genetic variants (13). Molecular methods provide more precise approaches of detection blood group antigens and help reduce the risk of alloimmunization. Real-time PCR (TaqMan) is widely used for this purpose, it has been used for genotyping different blood group systems, such as Rh, Kell, Duffy, Kidd, MNS, and Diego (13). Loop-mediated isothermal amplification (LAMP) determines ABO groups in under 30 min, obtained from non-blood samples such as saliva or dried blood (34). Multiplex PCR enables testing several alleles in a single run, while PCR-SSP is useful for detecting RHD variants and reliable PCR-based platform for routine detection of RHD variants in regions where DEL alleles are highly prevalent (35). More recently, next-generation sequencing (NGS) has been used for detailed profiling of both well-known and novel alleles. Whole-genome sequencing, used for the genetic analysis of variants associated with the Kidd, Duffy, and Kell blood group systems, was performed by generating 150 bp paired-end reads with 30X coverage depth employing Illumina technology, thus revealing all known and novel variants across the genome (29). These findings highlight the potential of NGS technologies to improve transfusion safety through more accurate and individualized blood group genotyping (29).

Transfusion management in multitransfused patients such as those with sickle cell disease (SCD) or thalassemia, is challenging because the high risk of alloimmunization. Conventional serological matching of antigens like C, E, and K, is not enough, since these patients commonly carry variant alleles. Molecular sequencing was used to identify RH allele frequencies of SCD patients and African American donors. The study revealed that 29% of RHD and 53% of RHCE alleles were altered in both groups. These genotypic differences contribute to alloimmunization, despite conventional matching. While practical barriers such as the high cost remain, these findings highlight the clinical importance of RH genotyping as a routine tool in transfusion practice to reduce alloimmunization risk in transfusion-dependent patients (36). Although RH genotyping and next-generation sequencing (NGS) offers promise tools for characterizing genetic variants and may in the future improve transfusion compatibility in chronically transfused patients, their ability to reduce alloimmunization has yet to be established and challenges such as cost and data complexity remain (36). In contrast, well-established interventions have already been used and demonstrated observable benefit. Universal leukoreduction of red blood units has been associated with decreased new alloantibody formation leading to significant reduction in alloimmunization rates (37). Similarly, implementation of ABO-identical platelets and cryoprecipitate have reduced both RBC alloimmunization and transfusion reactions (38). Comprehensive analyses highlight that leukoreduction, ABO-identical transfusion, and washing of blood products remain the most reliable and cost-effective approaches for reducing alloimmunization at present (39).

#### 2.6.3 Donor registry expansion and rare phenotype detection

Expanding donor registries and detecting rare phenotypes plays a pivotal role in transfusion medicine, as they directly impact the availability of antigen-matched blood for patients with alloimmunization or rare blood group. Microarrays-based genotyping has enabled large-scale screening of donors for extended antigen typing. For instance, the application of microarray genotyping in genetically diverse populations involved resolving complex blood group systems such as Rh and MNS, where highly homologous regions often complicate serological interpretation (40). The microarray platform validated against MALDI-TOF mass spectrometry achieved 99.95% concordance and a 99.65% call rate. It delivers high-throughput, accurate, and cost-effective genotyping, thus demonstrating its potential utility in routine donor screening, patient transfusion support, reduction of alloimmunization risk in multitransfused patients, and diversity-focused donor registry expansion (40).

#### 2.6.4 Detection of transfusion-transmitted pathogens

Pathogen detection represents another important application of molecular platforms in transfusion medicine. Implementation of nucleic acid testing (NAT) and PCR in transfusion medicine has significantly improved the safety of blood supplies. NAT allows detection of viral nucleic acids during the early window period, shortens the time to detection of infection and reducing the risk of transmitting infections such as human immunodeficiency virus (HIV), hepatitis B virus (HBV), and hepatitis C virus (HCV) (41). Another illustrative example is the development of DNA oligonucleotide microarray known as a pathogen chip that was designed to detect transfusion-transmitted RNA viruses via sequence-specific probe hybridization on a chip. Using 1,769 oligonucleotide probes, the chip identified 16 clinically relevant viruses, including HIV, HCV, HBV, dengue, Zika, and chikungunya in both individual and pooled plasma samples. This approach offers a valuable tool for comprehensive, rapid, and reproducible viral detection (42).

Table 1 summarizes the advances in molecular blood group diagnostics.

**Table 1:** Advances in molecular blood group diagnostics.

Technique	Mechanism	Clinical application	Example clinical conditions	Advantages	Disadvantages	References
PCR-based methods (PCR-RFLP, PCR-ASP, PCR-SSP, etc.)	Utilize designed primers specific to known alleles to amplify DNA fragments. The results are analyzed through gel electrophoresis or restriction enzyme digestion.	Used in genotyping of known polymorphisms in common blood group systems (e.g., Rh, Kell, Kidd). Able to distinguish between homozygous and heterozygous.	HDFN: Detects fetal RHD allele in RhD-negative mother. Autoimmune hemolytic anemia AIHA: confirms variant antigens that may evade serological typing. Transfusion: determines antigen profile in multitransfused patients Donor matching: Identifies donors with rare phenotypes	Specific for known polymorphisms. Cost-effective, and well-established.	Limited to predefined variants, low throughput.	(77, 78)
Real-time PCR (qPCR)	Real-time amplification and quantification of target DNA using fluorescent probes, more sensitive and faster than conventional PCR.	Enables non-invasive prenatal genotyping of fetal antigens (e.g., RhD, Kell) from maternal plasma.	HDFN: non-invasive detection of fetal RhD/KEL1 enabling early intervention of HDFN. Transfusion: Enabling rapid genotyping in patients with warm autoantibodies and overcomes the limitations of serological typing. Genotype clinically significant blood group antigens supporting extended antigen matching and reducing the risk of alloimmunization in transfusion recipients.	Faster results, quantitative analysis	Still limited to known polymorphisms	(79, 80)
Microarray-based genotyping	DNA is hybridized to immobilized oligonucleotide probes on chips; allowing detection of multiple SNPs simultaneously.	Screens for multiple blood group alleles in donors to find antigen-negative blood. Ideal for large-scale donor screening and complex antibody identification	Alloimmunization: resolving complex alloantibody profiles by enabling simultaneous identification of multiple blood group antigens. HDFN: identifies maternal zygosity or paternal genotype for predicting fetal risk. Rare donor registries: Identification of rare or extended phenotypes, supporting the development and maintenance of rare donor registries.	Simultaneous testing of multiple polymorphisms	High initial cost, complex data interpretation	(81)
Mass spectrometry (MALDI-TOF)	Determine mass-to-charge ratio of ionized DNA or protein fragments, the variants are distinguished based on mass differences.	Accurate identification of protein-level variants such RhCE and partial D antigen systems, which may not be reliably detected traditional serological methods.	Transfusion: detect weak or partial D variants that may be missed by serology, allowing appropriate antigen matching. Autoimmune hemolytic anemia (AIHA): Detect antigen mismatches that may lead to autoantibody production. Donor selection: Molecular level characterization of complex blood group antigens, minimizing the risk of transfusion reactions.	High accuracy, multiplex capability	Expensive equipment, complex sample preparation	(20, 81)
Next-generation sequencing (NGS)	High-throughput sequencing is used to analyze blood group gene loci.	Offer comprehensive blood group genotyping, by identifying rare or novel alleles.	Transfusion support in Sickle cell disease/thalassemia: full genotyping for extended antigen matching and may minimize alloimmunization. Population studies: identifying blood group allele frequencies and polymorphisms across different ethnic groups, thereby guiding the development of donor registries.	Detection of known and novel variants	High cost, complex data analysis	(81–83)
Long-read sequencing (Nanopore, PacBio)	Ability to generate long, continuous DNA sequence reads that span the entire blood group genes or haplotypes.	Identification of complex structural variants such as hybrid genes (e.g., RHD-CE-D), long-range haplotypes, or gene conversions causing genotype-serotype discrepancies in blood group systems.	Determination of complex Rh phenotypes. HDFN: Enables identification of hybrid or partial alleles that are often missed by short-read sequencing. Extended typing in alloimmunized patients: supports genotyping in individuals with complex serologic profiles or inconclusive molecular results.	Ability to resolve long haplotypes	Still in early stages of application	(84, 85)
CRISPR-Cas9 mediated gene editing	Gene-editing tool that uses guide RNA and Cas9/Cas13 enzyme, allowing the introduction of targeted insertions, deletions, or gene knockouts	Still experimental; used to engineer red blood cells devoid of immunogenic antigens, with the goal of generating universal donor cells.	Transfusion in alloimmunized patients: Produce antigen-negative RBCs. Enables engineering of antigen-negative red blood cells to overcome challenges associated with alloantibody.	Potential for” universal” donor cells.	Challenges in scalability and safety	(10)

## 3 Blood product engineering

### 3.1 Antigen modulation: RNA interference (RNAi) and microRNA (miRNA)

Blood group antigen expression holds significance in transfusion medicine, organ transplantation, and the understanding of disease susceptibility (43). The regulation of blood group antigen expression is determined by specific genes and their encoded glycosyltransferases (44).

Blood group antigens are found on the surface of RBCs and other cell types and are essential for immunological recognition (44). Obtaining knowledge on their expression and silencing and using techniques such as gene knockdown or knockout can significantly affect transfusion medicine (12). Blood group systems such as ABO, Rh, Kell, and Duffy are encoded by specific loci that impact glycoprotein and glycolipid synthesis on the cell surface (45). The ABO gene encodes glycosyltransferases, forming an ABO system (8). Genetic variations in this region, especially single nucleotide polymorphisms (SNPs), can affect enzyme activity and lead to variations in antigen expression levels (44, 46). For instance, alterations in the RHD gene, such as SNPs and hybrid alleles can alter antigen expression and give rise to weak D, partial D, or Del phenotypes (47).

Techniques like RNAi can assist in the selective suppression of the specific blood group antigens, for example, targeting the ABO gene to reduce its expression and lowering the risks linked with ABO incompatibility during transplantation (48, 49).

Moreover, overexpressing antigens such as ABO RhD in cell models helps in understanding the immunogenic and biochemical pathways involved in their synthesis (49). In addition, studies on ABH antigen regulation help explain the disappearance of ABH antigens during carcinogenesis and highlighted potential therapeutic targets in ABO-mismatched organ transplantation (45).

A pressing challenge in transfusion medicine is the availability of RBCs with extremely rare phenotypes (50), such as Rh-null (in which Rh antigens are absent from RBC membranes) (50). Rh-null blood cells are important for transfusion in patients with the same phenotype and for the diagnosis of Rh alloimmunization, especially in complicated cases like pregnancy (50).

### 3.2 iPSC-derived RBCs

Recent advances have focused on the production of RBCs from different cell sources. Human-induced pluripotent stem cells (hiPSCs) can be used as they offer an unlimited number of hemopoietic progenitor cells that can differentiate into erythroid cells (51), which can be produced from peripheral blood mononuclear cells and modified through gene editing, thus offering a novel system to produce customized red cells (51). Cells derived from hiPSCs could remedy the shortage in situations where rare blood types like Rh-null and Kell-negative phenotypes would provide greater compatibility for patients with alloimmunization (52, 53).

Induced pluripotent stem cells (iPSCs) have been generated from the dermal fibroblasts of an individual with a Bombay blood phenotype using Yamanaka factors (Oct4, Sox2, Klf4, and c-Myc). These iPSCs were established to be pluripotent and capable of significant cellular differentiation into various types. Furthermore, the hiPSCs maintained the genetic mutations associated with the Bombay phenotype, notably, the FUT1 and FUT2 gene mutations. Cells differentiated from hiPSCs can efficiently produce hematopoietic lineage cells positive for markers such as CD34 and Runx1, along with the erythroid markers *α*-globin and *γ*-globin. The abovementioned study represents a step toward the production of universal donor RBCs (53).

**Figure 1:**
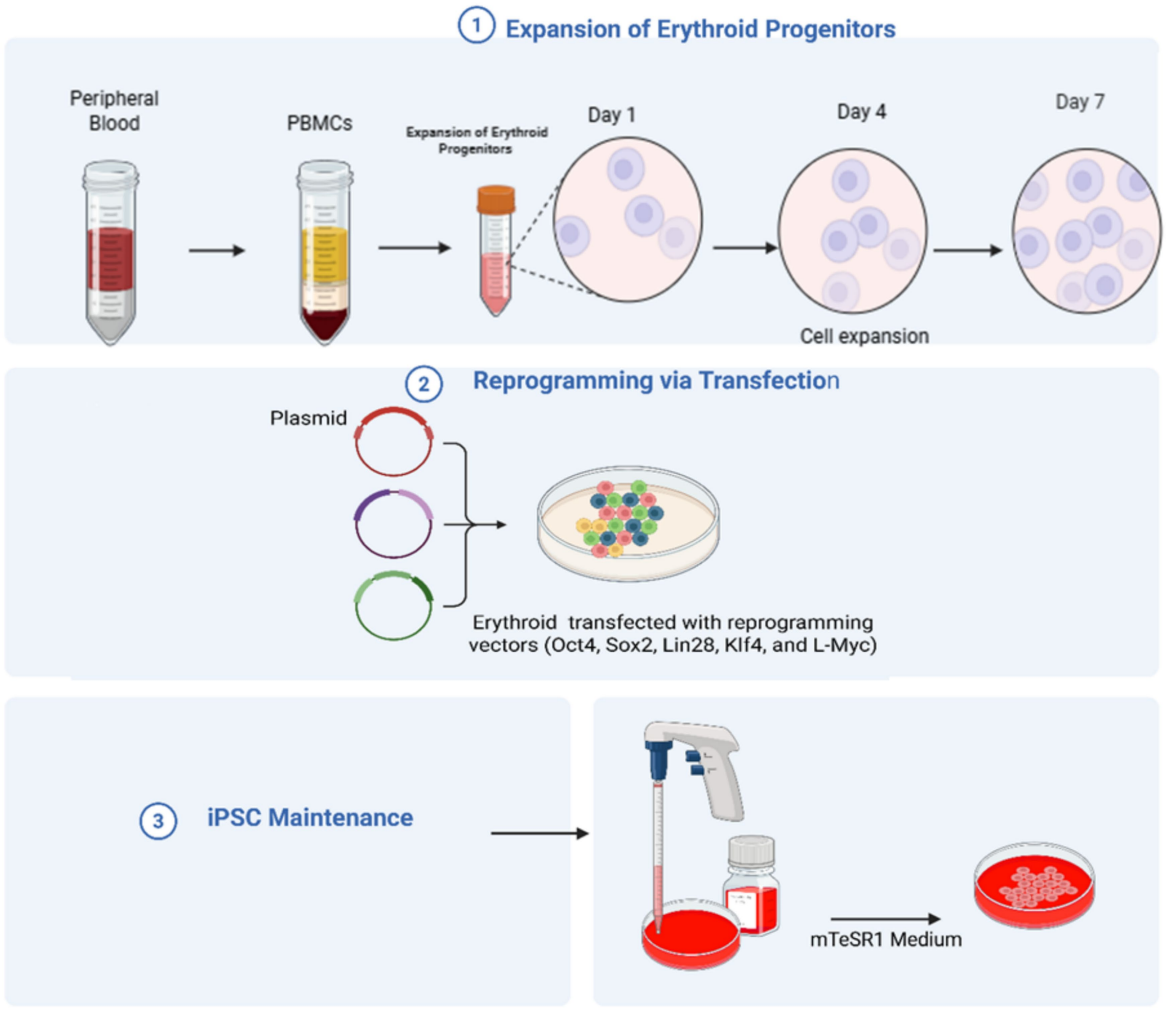
The production of human induced pluripotent stem cells from peripheral blood. Expansion of Erythroid Progenitors: First Peripheral blood is collected, then peripheral blood mononuclear cells (PBMCs) are isolated. These cells are cultured under specific conditions to promote the growth of erythroid progenitors. The progenitor cells then grow over several days (Day 1 → Day 4 → Day 7), producing enough cells for transfection. Reprogramming via transfection: The erythroid progenitors are then transfected with plasmids carrying (Oct4, Sox2, Lin28, Klf4, and L-Myc), promoting the formation of iPSC-like colonies. iPSC maintenance: the generated iPSCs are maintained in mTeSR1 medium to maintain pluripotency and self-renewal (created using BioRender.com).

Park et al. (54) established the generation of autologous iPSC-derived RBCs from peripheral blood mononuclear cells without chromosomal mutations, as shown in Figure 1. Another group of researchers also established hiPSCs from the primary bone marrow CD34 + cells of an O-negative blood donor (53). Table 2 summarizes these technologies and their usage.

**Table 2:** Therapeutic applications and clinical impact of molecular and cellular innovations in transfusion medicine.

Technology	Clinical use	Implementation status	Implementation status impact
Molecular genotyping	Extended phenotype matching, rare donor identification	Implemented in specialized centers	Improved matching for multitransfused patients, although this may result in difficulty locating compatible units
NGS-based blood group profiling	Comprehensive antigen matching	Emerging technology, limited clinical use	Potential for personalized transfusion strategies
Non-invasive fetal RHD genotyping	Prevention of Hemolytic Disease of the Fetus and Newborn (HDFN)	Implemented in some countries	Reduced need for invasive procedures, targeted prophylaxis
CRISPR-engineered RBCs	Potential for” universal” donor cells	Experimental stage	Could revolutionize transfusion for rare blood types
iPSC-derived diagnostic RBCs	Identification of complex Rh antibodies	Research stage	Improved diagnostics for patients with rare Rh types
Recombinant blood products	Treatment of coagulation disorders	Some products in clinical use	Reshaping the demand for plasma-derived products
Gene therapy	Treatment of hematologic diseases	Some are FDA approved and in use clinically	Potential for curative treatments

### 3.3 CRISPR gene-edited cells

#### 3.3.1 CRISPR-mediated antigen deletion for universal RBCs

The introduction of gene-editing technologies like CRISPR-Cas9 has revolutionized the arena of blood product development (50). Antigens from red cells can be deleted using these technologies to produce universal donor blood. Antigens from major blood group systems, such as the ABO, Rh, Kell, Duffy, and GPB systems, have been successfully deleted. These advances will go a long way in lowering immunogenicity during transfusions (10).

**Figure 2:**
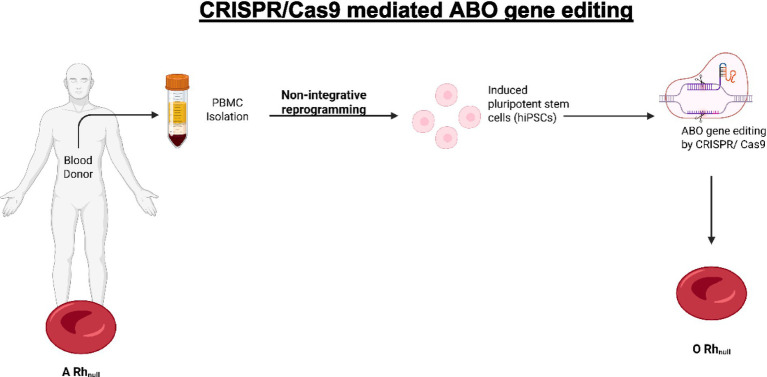
CRISPR/Cas9 mediated ABO gene editing allows the conversion of blood type (created using BioRender.com).

An innovative application of CRISPR/Cas9 involves the conversion of blood type A to blood type O in Rh-null donor-derived hiPSC lines; this is seen as a pathway for producing universally compatible red cells from donors with rare blood types (55). The authors targeted the ABO gene to disrupt the enzymatic activity responsible for antigen A synthesis using two approaches: (1) introducing a specific mutation (c.261delG) prevalent in type O individuals and (2) creating an incipient knockout of the ABO gene. Both approaches were successful in editing the ABO gene without compromising the pluripotence or potential for differentiation of the stem cells, as shown in Figure 2 (55).

#### 3.3.2 Generation of multi-antigen knockout erythroid cells

Hawksworth et al. (10) improved transfusion compatibility by editing erythroblasts using CRISPR to eliminate the expression of antigens while maintaining cell functionality. They generated a human erythroblast cell line called BEL-A using CRISPR-Cas9 genome editing. These researchers were attempting to improve ABO-compatible RBC transfusions by knocking out specific blood group genes, with the targeted antigens belonging to the ABO, Rh, Kell, Duffy, and MNS blood groups, thereby creating customized RBC phenotypes via bi-allelic knockouts (10). This allowed for the generation of erythroblast cell lines capable of differentiating into functional reticulocytes with a complete deficiency of the targeted antigens. Through multiple gene knockouts, the authors generated a cell line capable of developing into reticulocytes, which are highly transfusion-compatible (10). However, other studies have highlighted the increased immunogenic risks associated with reticulocytes and these immature cells persisting both during storage and in the recipient’s circulation after transfusion (56, 57). Therefore, while multi-antigen knockout strategies represent exciting innovation, their clinical application will require ensuring full maturation into enucleated red cells (10, 56).

#### 3.3.3 CRISPR-based development of RhD-negative blood

Another remarkable achievement is the development of RhD-negative blood using CRISPR/Cas9 technology. The RHD gene was targeted by a homology-directed repair (HDR)-based CRISPR/Cas9 system. This system mediated the introduction of a premature stop codon in the RHD gene, knocking down its expression in hiPSCs derived from human umbilical arterial endothelial cells. This source was chosen for its high hematopoietic differentiation potential. Following genetic modification, RHD knockout hiPSCs were induced to differentiate into erythrocytes using an optimized four-phase protocol involving stepwise changes in oxygen conditions to mimic natural hematopoietic environments (10). The erythrocytes obtained expressed standard RBC markers (CD71 and CD235a) but lacked RhD antigen expression. Functional tests, including agglutination tests, confirmed that the modified erythrocytes did not agglutinate with anti-RhD antibodies, thus demonstrating an RhD-negative phenotype. This work represents a significant step toward creating universal donor blood products for transfusion medicine (10).

#### 3.3.4 HLA-universal platelets and megakaryocytes

Another noteworthy achievement is the engineering of HLA-universal megakaryocytes and platelets to reduce the risk of alloimmune platelet refractoriness, mainly in patients who require frequent platelet transfusions (58). The elimination of HLA antigens on platelets has opened possibilities for better compatibility and fewer immune response risks during platelet transfusion therapies (59–61).

### 3.4 miRNA-based therapeutics in transfusion and transplantation

miRNAs have been recognized as vital modulators of blood group antigens (62). miRNAs are small, non-coding RNAs used for post-transcriptional gene regulation that regulate the expression of blood group antigens on RBCs and other cell types (63). In transfusion medicine, miRNA-based therapeutic approaches have been studied to reduce the risks of alloimmunization and enhance compatibility (64, 65). Kronstein-Wiedemann et al. (12) determined the role of miRNAs in the regulation of the expression of ABO blood group antigens during erythropoiesis. They found that miR-331-3p and miR-1908-5p directly regulate glycosyltransferase A and B mRNA, as increasing expression of these miRNAs in hematopoietic stem cells significantly reduced A and B antigen expression on RBCs (12, 45). The Rhesus protein RhAG is regulated by miR-9. RhAG was one among 170 putative target genes with an inverse correlation with miR-9 expression of miR-9 (66). The expression of the KLF1 protein was also downregulated upon the overexpression of miR-326 and increased expression of *γ*-globin. This decreased the amounts of Lutheran and other blood group antigens (the Indian, P1PK, Landsteiner-Wiener, Knops, OK, RAPH, and I blood group systems) (67). In autoimmune hemolytic anemia, silencing miRNAs involved in autoantibody production showed potential in the reduction of hemolysis (68). Moreover, downregulation of the miRNAs that target RhD expression may reduce the immune response in sensitized Rh-negative individuals, resulting in better transfusion outcomes (12).

In transplantation, miRNA-based interventions have been used to control the expression of ABO and HLA antigens on donor tissues to reduce organ rejection risk (68). For example, silencing miR-150, which directly regulates erythropoiesis, was found to reduce immunogenic antigen levels, thus reducing the immune reaction (69, 70). Another example is miR-223, whose effects on HLA antigen expression on megakaryocytes could reduce HLA-mediated alloimmune responses and thereby improve the compatibility of platelet transfusions for patients suffering refractory thrombocytopenia (58, 71). Silencing miR-181a in transplantation models reduces the rates of graft rejection by decreasing the immunogenicity of transplanted tissues. This miRNA has been shown to regulate HLA-DR antigen expression on donor tissues (72). miR-451 is involved in erythrocyte maturation and the expression of blood group antigens. Its downregulation may improve the survival and compatibility of transfused RBCs by minimizing the effect of immune-mediated clearance (73).

## 4 Conclusion

Understanding the molecular basis of blood group antigens and their associated technologies has driven a revolution in transfusion medicine and transplantation and led to a better understanding of disease susceptibility (8). In the last few decades, the understanding of blood group systems has progressed substantially: more than 36 systems, along with 300 antigens, have been described by the International Society of Blood Transfusion (74). Clinically significant antigens are essential in determining transfusion and transplantation compatibility (75). Molecular diagnostics, such as PCR and NGS, have been used to enhance blood group typing accuracy and aid in potentially reducing the risk of alloimmunization (76). Such methods may resolve the challenges associated with traditional serological methods (by identifying rare phenotypes) and address serological discrepancies (76). Gene editing technologies, such as CRISPR-Cas9, have been used to develop universal donor cells and enhance the engineering of blood cells (50). Their application in silencing the expression of immunogenic antigens, such as RhD and Kell, holds great promise for allo-immunized patients by reducing severe transfusion complications (50). Despite these developments, a huge gap remains between such advances and their application in clinical practice (52). The complexity and high costs of molecular diagnosis and gene editing make these innovations inaccessible in many parts of the world (52).
